# Synthesis and anti-inflammatory activities of two new N-acetyl glucosamine derivatives

**DOI:** 10.1038/s41598-024-61780-2

**Published:** 2024-05-14

**Authors:** Zhichang Zhang, Weicheng Wang, Peng Xu, Quanjun Cui, Xinlin Yang, Ameer E. Hassan

**Affiliations:** 1 Department of Orthopaedic Surgery, School of Medicine, University of Virginia, 450 Ray C. Hunt Drive, Charlottesville, VA 22903 USA; 2 Department of Pathology, University of Virginia, Charlottesville, 22903 USA; 3 Department of Orthopaedic Surgery, The First Affiliated Hospital of Xinxiang Medical University, Weihui, 453100 Henan China; 4 Department of Neuroscience, Valley Baptist Medical Center, 2101 Pease St., Harlingen, TX 78550 USA

**Keywords:** N-acetyl glucosamine, Bi-deoxygenation, Inflammation, Macrophages, Diseases, Medical research, Molecular medicine

## Abstract

N-acetyl glucosamine (NAG) is a natural amino sugar found in various human tissues with previously described anti-inflammatory effects. Various chemical modifications of NAG have been made to promote its biomedical applications. In this study, we synthesized two bi-deoxygenated NAG, BNAG1 and BNAG2 and investigated their anti-inflammatory properties, using an in vivo and in vitro inflammation mouse model induced by lipopolysaccharide (LPS). Among the parent molecule NAG, BNAG1 and BNAG2, BNAG1 showed the highest inhibition against serum levels of IL-6 and TNF α and the leukocyte migration to lungs and peritoneal cavity in LPS challenged mice, as well as IL-6 and TNF α production in LPS-stimulated primary peritoneal macrophages. BNAG2 displayed an anti-inflammatory effect which was comparable to NAG. These findings implied potential application of these novel NAG derivatives, especially BNAG1, in treatment of certain inflammation-related diseases.

## Introduction

Inflammation is a defense mechanism by which the body's immune system responds to an injury, infection, or irritation. Inflammation can happen in either a short (acute) or long (chronic) term. In the presence of the acute inflammation, various chemicals/cytokines are released and the immune cells such as macrophages and lymphocytes are transported to the affected area, leading to symptoms including redness, swelling, heat, and pain^1^. On the other hand, the chronic inflammation can contribute to many diseases, such as ischemic heart disease, stroke, cancer, diabetes mellitus, chronic kidney disease, non-alcoholic fatty liver disease, neurodegenerative disease and Inflammatory arthritis. These chronic inflammatory diseases have been regarded as the most significant cause of world deaths^2,3^.

Natural products are organic compounds obtained from living organisms such as insects, plants, animals, humans, marine, and microorganisms. A lot of studies have demonstrated the positive effects of natural products in treating inflammation-related diseases^3,4^. N-acetyl glucosamine (NAG), an acetylated derivative of glucosamine, is an essential component of bacterial and fungal cell walls as well as various human tissues^5^. Due to its low cost, ready availability and less side effects as a natural product and significant anti-inflammatory activity, NAG has attracted much attention as an ideal candidate for the treatment of inflammation-related diseases such as joint damage^6,7^, inflammatory bowel disease^8^, autoimmune diseases^9^, and viral respiratory infections^10,11^. In particular, many clinical trials on NAG have been performed to treat patients with joint disorders, including arthritic diseases, osteoarthritis, rheumatoid arthritis, cartilage damage, joint injury and degenerative joint disease^5^.

Chemical modification of NAG can be performed to introduce specific functional groups or alterations to its structure, thereby modulating its properties to promote its biomedical applications. Lee et al. reported that acetylation of NAG improved its cell membrane permeability, leading to a greater capacity to inhibit T-helper 1 (TH1)/TH17 responses and autoimmunity^12^. Morrison et al. demonstrated that among several NAG analogues, the 6-fluoro and 6-deoxy derivatives were potent inhibitors of Poly‑N‑acetylglucosamine polymerization and biofilm formation in a *E. coli* strain transformed by a specific NAG kinase which provided a non-native salvage pathway to take use of these NAG analogues^13^. Based on the report of Morrison et al., it is interesting to investigate if the location and number of deoxygenation will influence the biological activities of NAG. As the first step of this investigation, two 4,6-bideoxy-n-acetyl-D-glucosamine (BNAG), including BNAG1 (1,3-bideoxy-n-acetyl-D-glucosamine or 2-Acetamido-1,2,3-trideoxy-D-glucose) and BNAG2 (4,6-bideoxy-n-acetyl-D-glucosamine or 2-Acetamido-2,4,6-trideoxy-D-glucose), were successfully synthesized. In addition, the anti-inflammatory activities of BNAG1 and BNAG2 against lipopolysaccharide (LPS)-induced inflammation in mice and in vitro primary peritoneal macrophages were determined, with the parental molecule NAG as control.

## Results

### Synthesis and characterization of BNAG1 and BNAG2

**Figure 1:**
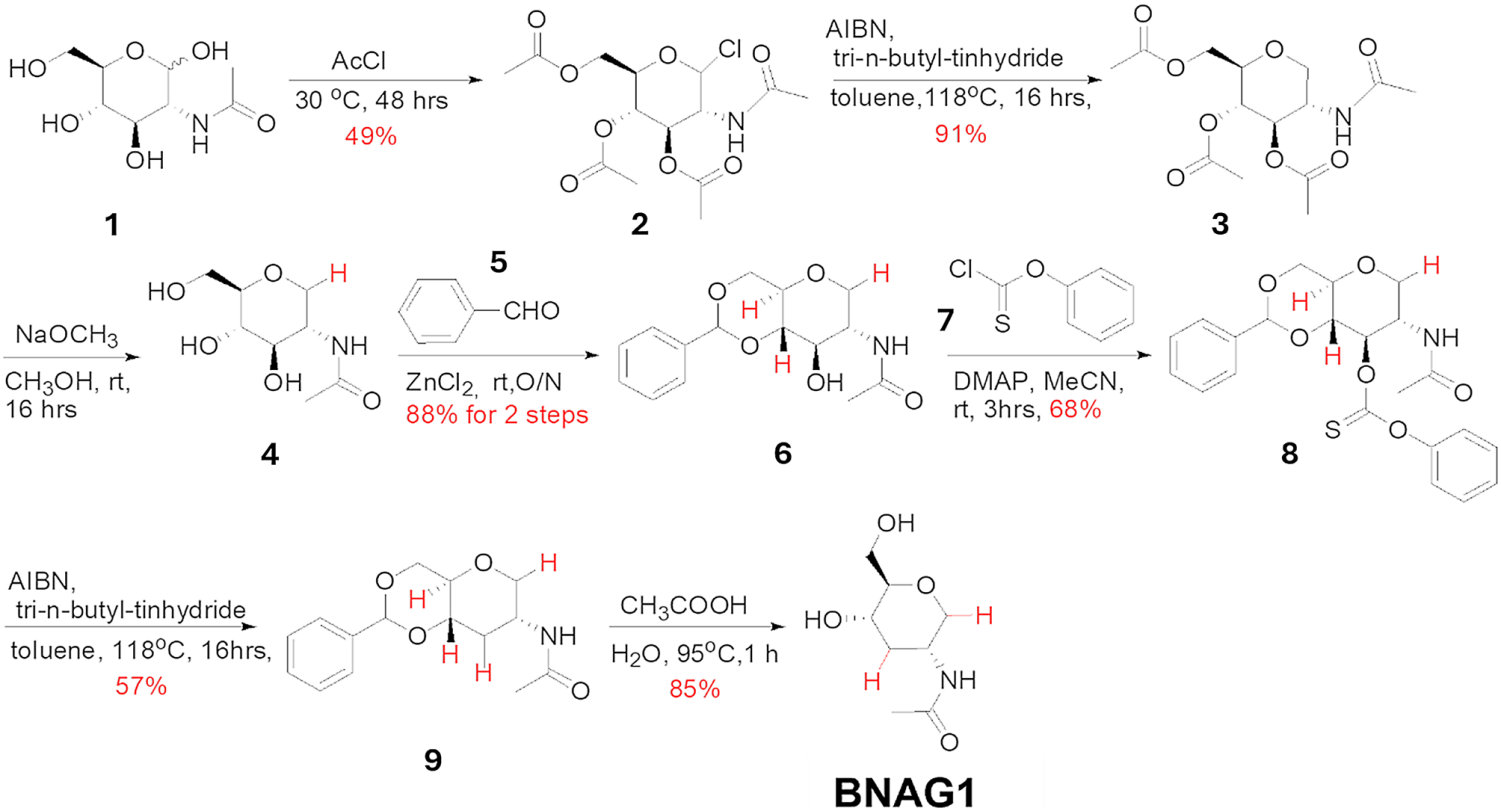
Synthetic route of BNAG1.

**Figure 2:**
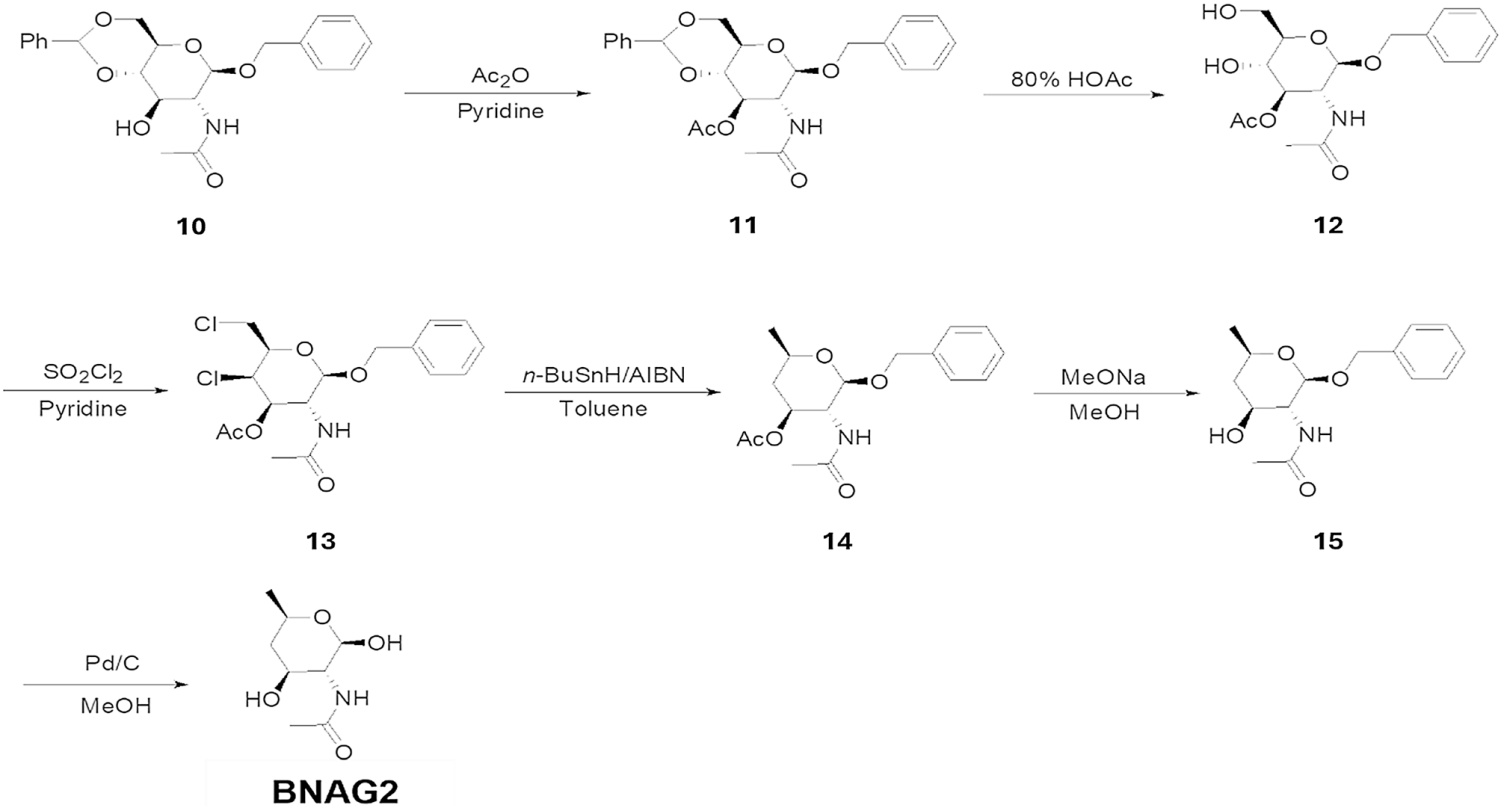
Synthetic route of BNAG2.

As illustrated in Figs. 1 and 2, BNAG1 and BNAG2 (each purity > 95%) were obtained after multiple chemical reactions, with the starting materials of NAG and 1, 4, 6-hydroxyl protecting NAG, respectively. The characterization data of the key compounds for two NAG derivatives were as follows:

### BNAG1:

**2**: LC–MS: 366.25 [M + 1] ^+^. ^1^H NMR (400 MHz, CDCl_3_) δ 6.17 (d, *J* = 3.7 Hz, 1H), 5.87 (d, *J* = 8.7 Hz, 1H), 5.31 (dd, *J* = 10.7, 9.4 Hz, 1H), 5.19 (t, *J* = 9.7 Hz, 1H), 4.56 – 4.47 (m, 1H), 4.31 – 4.21 (m, 2H), 4.15 – 4.07 (m, 1H), 2.08 (s, 3H), 2.03 (s, 6H), 1.97 (s, 3H).

**3**: LC–MS: 332.25 [M + 1]^+^. ^1^H NMR (400 MHz, CDCl_3_) δ 5.66 (d, *J* = 7.2 Hz, 1H), 5.07 (t, *J* = 9.5 Hz, 1H), 4.93 (t, *J* = 9.7 Hz, 1H), 4.17 (m, 4H), 3.57 – 3.49 (m, 1H), 3.15 (m, 1H), 2.10 – 2.02 (m, 9H), 1.93 (s, 3H).

**4**: ^1^H NMR (400 MHz, CD_3_OD) δ 3.89 (dd, *J* = 10.9, 5.2 Hz, 1H), 3.84 – 3.75 (m, 2H), 3.60 (m, 1H), 3.38 – 3.32 (m, 1H), 3.24 (d, *J* = 8.7 Hz, 1H), 3.14 (m, 1H), 3.09 (t, *J* = 10.9 Hz, 1H), 1.94 (s, 3H).

**6**: LC–MS: 294.20 [M + 1] ^+^. ^1^H NMR (400 MHz, CD_3_OD) δ 7.53 – 7.43 (m, 2H), 7.39 – 7.26 (m, 3H), 5.57 (s, 1H), 4.26 – 4.18 (m, 1H), 3.97 – 3.88 (m, 2H), 3.77 – 3.62 (m, 2H), 3.48 (d, *J* = 9.1 Hz, 1H), 3.33 (d, *J* = 5.0 Hz, 1H), 3.21 (s, 1H), 1.95 (s, 3H).

**8**: LC–MS: 430.2 [M + 1] ^+^, 452.2 [M + Na] ^+^. ^1^H NMR (400 MHz, DMSO-*d*_6_) δ 7.42 – 7.33 (m, 7H), 7.27 (s, 1H), 7.00 (d, *J* = 7.9 Hz, 2H), 5.68 (s, 1H), 4.26 – 4.19 (m, 2H), 3.93 (s, 1H), 3.81 (dd, *J* = 11.3, 5.7 Hz, 1H), 3.74 (s, 1H), 3.48 (d, *J* = 11.3 Hz, 2H), 1.80 (s, 3H).

**9**: LC–MS: 278.25 [M + 1] ^+^. ^1^H NMR (400 MHz, CD_3_OD) δ 7.43 (m, 2H), 7.32 (m, , 3H), 5.58 (s, 1H), 4.20 (m, 1H), 4.05 (m, 1H), 3.98 – 3.91 (m, 1H), 3.68 (t, *J* = 10.4 Hz, 1H), 3.63 (d, *J* = 1.8 Hz, 1H), 3.26 – 3.19 (m, 1H), 3.12 (t, *J* = 10.8 Hz, 1H), 2.30 – 2.22 (m, 1H), 1.91 (s, 3H), 1.58 (q, *J* = 11.6 Hz, 1H).

**BNAG1**: LC–MS: 190.20 [M + 1] ^+^. ^1^H NMR (400 MHz, CD_3_OD) δ 3.94 – 3.79 (m, 3H), 3.61 – 3.52 (m, 1H), 3.48 – 3.41 (m, 1H), 3.05 – 2.95 (m, 2H), 2.25 – 2.18 (m, 1H), 1.90 (s, 3H), 1.37 (q, *J* = 11.7 Hz, 1H). ^13^C NMR (100 MHz, DMSO-d6): δ 169.3, 83.4, 69.64, 65.0, 61.9, 45.2, 22.8. Anal. Calcd for C_8_H_15_NO_4_: C, 50.78; H, 7.99; N, 7.40. Found: C, 50.58; H, 7.88; N, 7.21. [α]^25^_D_ 4.88° (c = 1.0, methanol).

### BNAG2:

**BNAG2**: LC–MS: 190.20 [M + H]^+^. ^1^H NMR (400 MHz, DMSO-*d*_*6*_): *δ* 7.55 (s, 1 H), 6.24 (s, 1 H), 4.89 (s, 1 H), 4.54 (s, 1 H), 3.98–3.95 (m, 1 H), 3.66–3.62 (m, 1 H), 3.45–3.40 (m, 1 H), 1.87–1.83 (m, 1 H), 1.79 (s, 3 H), 1.13–1.07 (m, 1 H) 1.03 (s, 3 H). ^13^C NMR (100 MHz, DMSO-d6): δ 170.0, 91.7, 64.4, 62.8, 56.3, 42.9, 23.2, 21.5. Anal. Calcd for C_8_H_15_NO_4_: C, 50.78; H, 7.99; N, 7.40. Found: C, 50.60; H, 8.24; N, 7.32. [α] ^25^_D_ -23.8° (c = 1.0, methanol).

### In vivo experiments

#### Serum levels of inflammatory cytokines

**Figure 3:**
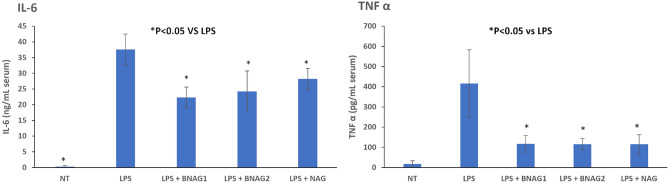
The serum IL-6 and TNF α levels in mice with various treatments including non-treatment (NT), LPS alone, LPS + BNAG1, LPS + BNAG2 and LPS + NAG. No drugs were added to NT group. LPS: 10 mg/kg, intraperitoneal injection; BNAG1, BNAG2 & NAG: 300 mg/kg, intravenous injection. N = 8.

**Figure 4:**
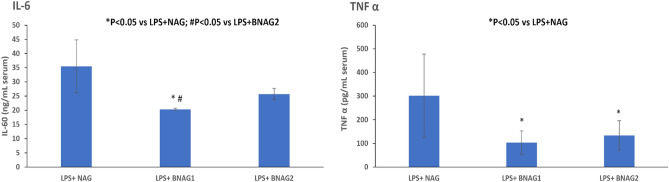
The serum IL-6 and TNF α levels in mice with various treatments including LPS + NAG, LPS + BNAG1 and LPS + BNAG2. LPS: 10 mg/kg, intraperitoneal injection; BNAG1, BNAG2 & NAG: 200 mg/kg, intravenous injection. N = 8.

The production of inflammatory cytokines coordinates the response to infectious agents through the activation and recruitment of immune cells. To determine the production of inflammatory mediators, serum concentrations of IL-6 and TNF α were quantified. LPS increased serum levels of IL-6 and TNF α, and all three tested compounds BNAG1, BNAG2 and NAG at the dose of 300 mg/kg were able to significantly decrease serum levels of IL-6 and TNF α in LPS-mice (Fig. 3). There was no significant difference in their effects among the three compounds at this dose. However, BNAG1 showed significantly higher inhibitory activities against IL-6 and TNF α production than either NAG or BNAG2 at a reduced dose of 200 mg/kg (Fig. 4).

#### Leukocytes migration to the lung and the peritoneal cavity

**Figure 5:**
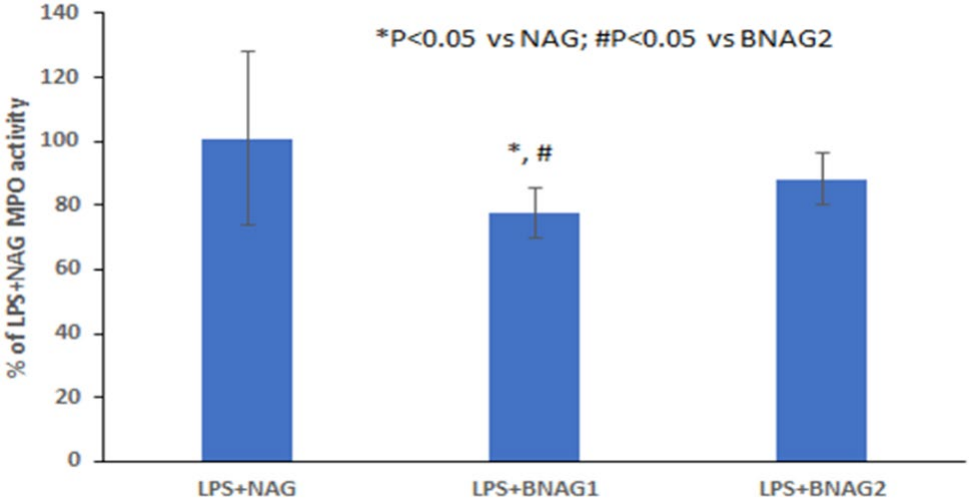
MPO activities of the Lung tissues from mice with various treatments including LPS + NAG, LPS + BNAG1 and LPS + BNAG2. LPS: 10 mg/kg, intraperitoneal injection; BNAG1, BNAG2 & NAG: 200 mg/kg, intravenous injection. N = 8.

**Figure 6:**
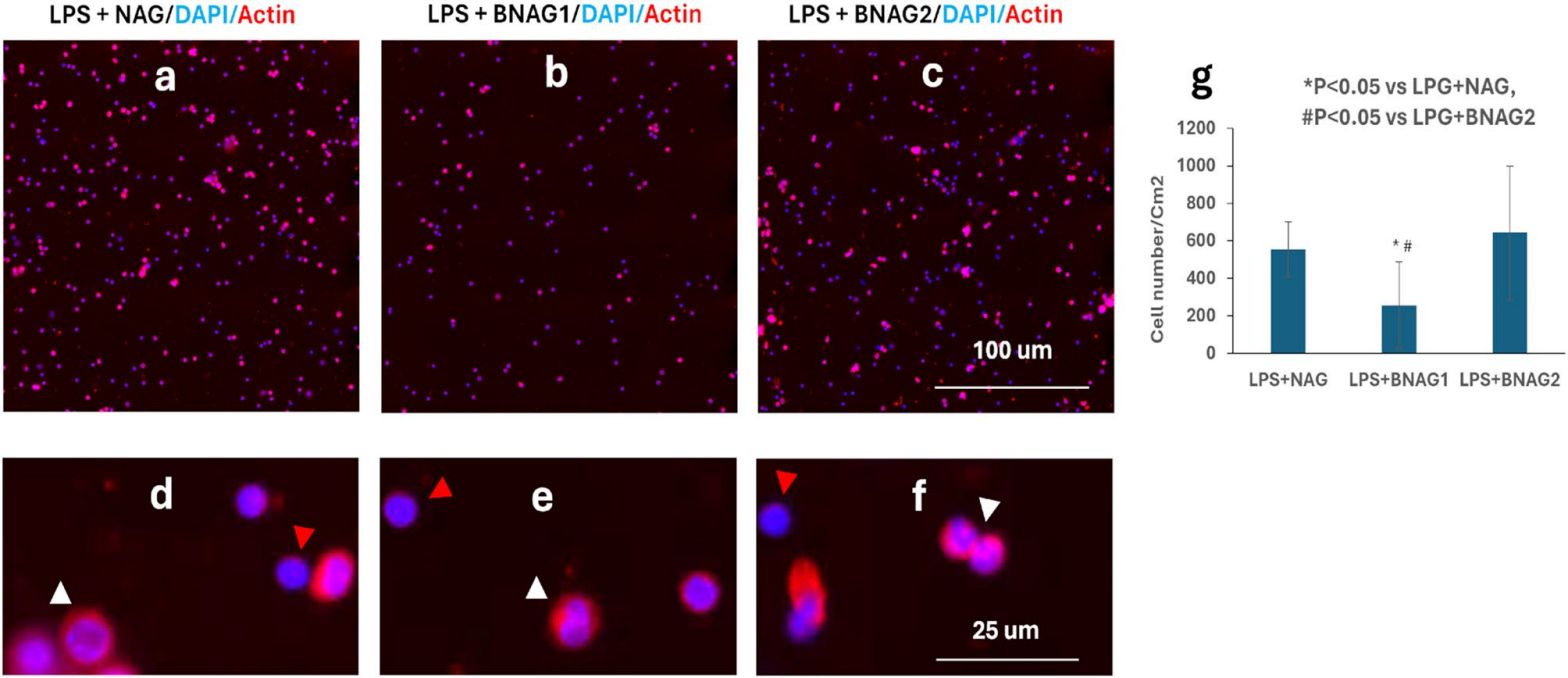
Leukocytes migration to the peritoneal cavity from mice with various treatments including LPS + NAG, LPS + BNAG1 and LPS + BNAG2. (**a**-**f**) Cells were double-stained with ALEXA FLUO 568 phalloidin/DAPI at low (**a**-**c**) and high (**d**-**f**) magnification. (**g**) Quantitative analysis of cell number in images (**a**, **b** and **c**). White arrowhead: neutrophils; Red arrowhead: monocytes. LPS: 10 mg/kg, intraperitoneal injection; BNAG1, BNAG2 & NAG: 200 mg/kg, intravenous injection. N = 8.

**Figure 7:**
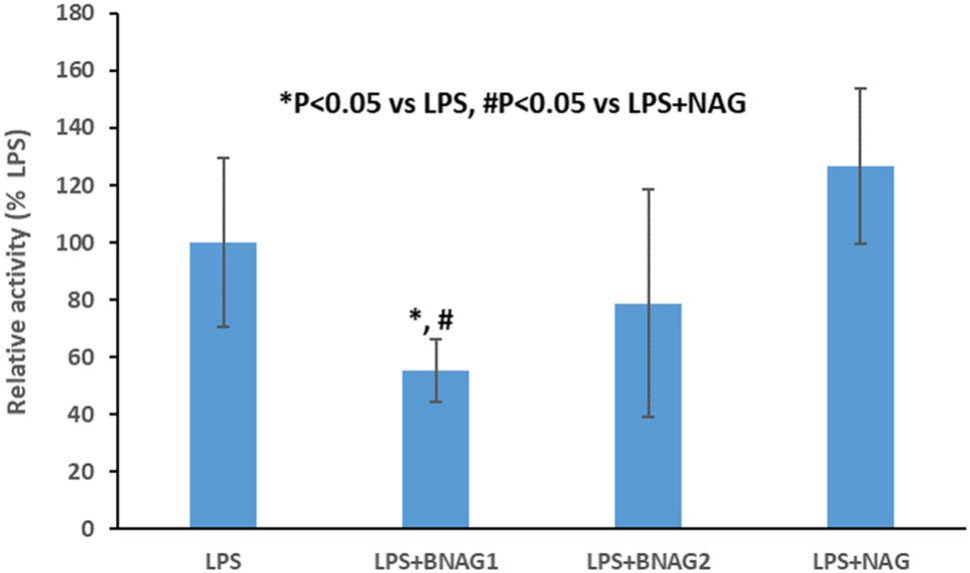
The WST-1 assay of peritoneal cells isolated from mice with various treatments including LPS alone, LPS + NAG, LPS + BNAG1 and LPS + BNAG2. LPS: 10 mg/kg, intraperitoneal injection; BNAG1, BNAG2 & NAG: 300 mg/kg, intravenous injection. N = 8.

Sequestration of leukocytes from the circulation is an event that can compromise an appropriate response to infection. To compare the effects of BNAG1 and BNAG2 with NAG on leukocytes infiltration in the lungs, the myeloperoxidase (MPO) activity of the lung tissues was determined. Figure 5 demonstrates that BNAG1 is the most effective in reducing leukocytes sequestration in the lung of LPS mice among the three compounds tested. To assess the migration of leukocytes to peritoneal cavity, cells harvested from peritoneal cavity were double-stained by the fluorescent dyes Alexa Fluo® 568 phalloidin and DAPI (Fig. 6a-f). Boundaries of the nucleus and cells could be readily viewed by the blue DAPI fluorescence and red Alexa Fluo® 568 fluorescence, respectively^14^. Both polymorphonuclear neutrophils with multilobed nucleus (white arrowhead) and monocytes (red arrowhead) were found in the images with high magnification (Fig. 6d-f). Quantification of cells based on the nucleus staining in each sample indicates that the LPS + BNAG1 group has the lowest amount of cells among the three groups including LPS + NAG, LPS + BNAG1 and LPS + BNAG2 (Fig. 6g). In addition, the peritoneal cells harvested from mice of four different groups (LPS alone, LPS + NAG, LPS + BNAG1 and LPS + BNAG2) were seeded on a 96 well plate, cultured for 24 h and tested by the WST-1 assay. It was found that the OD value of LPS + BNAG1 group was significantly lower than LPS alone group, while neither NAG nor BNAG 2 was (Fig. 7).

### In vitro experiments

**Figure 8:**
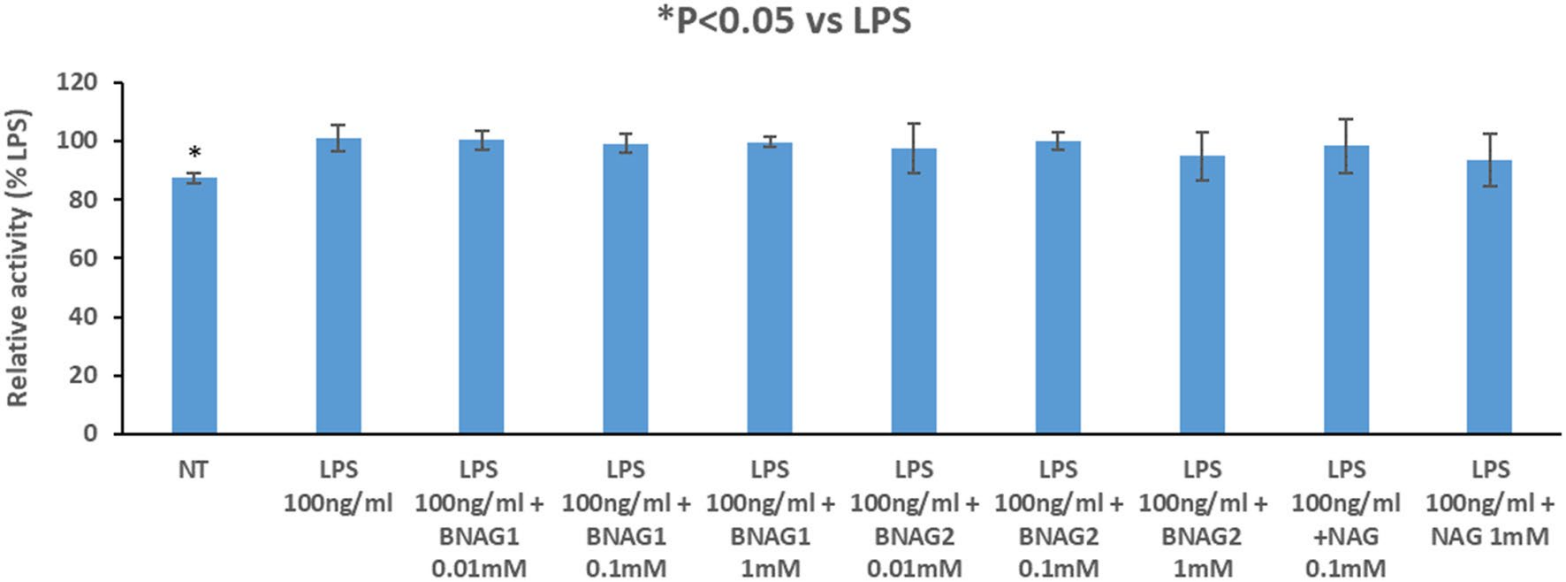
The effects of BNAG1, BNAG2 and NAG on primary mouse peritoneal macrophages determined by the WST-1 assay. Concentrations up to 1 mM were tested for each drug. No drugs were added to the non-treatment group (NT). There were eight repeats in each group (n = 8). *P < 0.05 vs LPS group. The experiment was duplicated.

**Figure 9:**
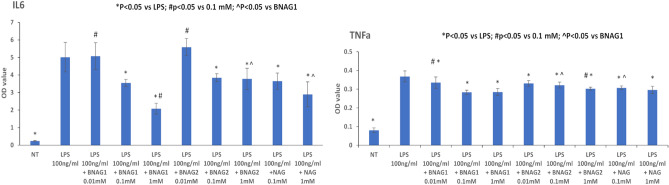
The ELISA tests for effects of BNAG1, BNAG2, and NAG at various concentrations on IL-6 and TNF α production in primary mouse peritoneal macrophages. There were eight repeats in each group (n = 8). The experiment was duplicated.

For in vitro assays, primary peritoneal macrophages were harvested from mice, cultured on a 96 well plate, and treated with 100 ng/mL LPS for 24 h with or without NAG, BNAG1 or BNAG2 at different doses of up to 1 mM. The non-treatment (NT) group was used as a negative control. Then the WST-1 assay and ELISA tests were performed to cells and culture medium, respectively. The WST-1 data showed that LPS caused a significant increase in cell OD value compared with NT, while LPS + BNAG1, LPS + BNAG2 or LPS + NAG at all doses didn’t induce significant change in cell OD value compared with LPS alone (Fig. 8). The ELISA tests demonstrated that LPS significantly stimulated the IL-6 and TNF α production, and NAG, BNAG1 and BNAG2 could significantly reduce IL-6 and TNF α levels elevated by LPS. Notably, BNAG1 was the most effective among them (Fig. 9).

## Discussion

NAG and its derivates have been reported in dietary supplements and for therapeutic development^5^. In the present study, we completed synthesis of two NAG derivatives, BNAG 1 and BNAG2. BNAG 1 was synthesized by 1,3-deoxygenation of NAG, while BNAG 2, on the other hand, was the product after 4,6-deoxygeneration of NAG. They were characterized by LC–MS, ^1^H NMR, ^13^C NMR and elemental analysis, and all analytical data confirmed the proposed chemical structures of BNAG1 and BNAG2, respectively. Furthermore, tri-n-butyltin hydride was used as a source of hydrogen atoms in the middle of the synthetic routes of both compounds, tin residues were tested by atomic absorption spectroscopy and found less than 0.01% in both final products. The maximum permissible levels of tin in food are typically 250 mg/kg for solid foods and 150 mg/kg for beverages^15^. Considering that NAG products were typically recommended via oral administration for an adult at a dose of 20 mg/kg/day (an equivalent amount of less than 0.2 mg/kg/day tin in BNAGs)^10^, it is concluded that BNAGs would be very safe agents as either dietary supplements or medical drugs. To our knowledge, only several chemical modifications of NAG have been published, including replacement of 6-hydroxyl by deoxy, fluoro, thiol group or amino substituents^13^, functionalization of 1-hydroxyl to produce a library of 2-acetamido-2-deoxy-D-glucono-1,5-lactone arenesulfonylhydrazones^16^, as well as single or multiple acetylation^12^. As such, BNAG1 and BNAG2 were the bi-deoxygenated derivatives of NAG reported for the first time.

Due to its technical ease and high reproducibility, LPS-induced mouse systemic inflammatory model is extensively used for anti-inflammatory assays of candidate drugs, including glucosamine^17,18^. In a study of Silva et al., mouse systemic inflammatory response was induced by intraperitoneal injection of LPS at two doses, 10 mg/kg and 20 mg/kg, and intravenous administration of 300 mg/kg glucosamine was able to inhibit the activity of LPS at each dose^17^. In the present experiment, 10 mg/kg LPS caused a significant increase in serum levels of IL-6 and TNF α (Fig. 3), indicating that the current LPS model was effective. Furthermore, all three compounds NAG, BNAG1 and BNAG2 displayed significant inhibitory effect on the elevated serum levels of cytokines IL-6 and TNF α production caused by LPS, however, no difference was found among these compounds at the tested dose of 300 mg/kg (Fig. 3). We suspected that 300 mg/kg was too high to distinguish the activities of these compounds from each other. According to a previous report where 200 mg/kg was chosen for glucosamine^18^, a lower dose of 200 mg/kg was applied to further test our compounds. Because the effectiveness of the LPS model was proven by the outcomes shown in Fig. 3, in the following assays we emphasized on three groups LPS + NAG, LPS + BNAG1 and LPS + BNAG2, minimalizing the number of animals. As anticipated, significant difference in anti-inflammatory activities appeared among the three compounds at 200 mg/kg. BNAG 1 exhibited the strongest inhibition against serum pro-inflammatory cytokine levels (Fig. 4), as well as leukocyte migration to the lung and the peritoneal cavity (Figs. 5, 6), among the three compounds at the dose of 200 mg/kg. The WST-1 assay with isolated peritoneal cells showed the lowest OD value of LPS + BNAG1 among the three groups, providing supportive evidence that BNAG1 was the most powerful compound for inhibition of leukocyte migration and/or superoxide production of peritoneal cells (Fig. 7). It is worth noting that the serum levels of pro-inflammatory cytokines obtained from our experiments were not exactly the same as those reported previously by other groups, but the trend was almost the same for the various treatments^17,18^. This might be due to different sources and operations of animals, isolated cells, LPS, and assay kits.

The anti-inflammatory properties of BNAG1 and BNAG2 with their parent molecule NAG were also compared using a well-established in vitro inflammation model of mouse primary peritoneal, induced by LPS^19,20^. The experiments revealed that BNAG1, BNAG2 and NAG at the tested doses caused few changes in cell proliferation/superoxide production of LPS-activated macrophages (Fig. 8). However, BNAG1 was found the most effective to reverse IL-6 and TNF α production stimulated by LPS among three compounds (Fig. 9), in accordance with our earlier report that BNAG1 was the most effective in the anti-inflammatory activity among the three compounds in LPS-activated mouse macrophage cell line RAW264.7^21^.

It has been well established that O-linked β-N-acetylglucosamine (O-GlcNAc), a post-translational protein modification, plays a crucial role in the regulation of various cellular processes, including signal transduction, transcription, protein degradation, and cellular stress response^22^. A single pair of enzymes, β-N-acetylglucosaminyl transferase (O-GlcNAc transferase, OGT) and β-N-acetylglucosaminidase (O-GlcNAcase, OGA), are involved in regulation of the dynamic cycling of this protein modification. OGT transfers the GlcNAc moiety from uridine diphosphate N-acetylglucosamine (UDP-GlcNAc) to the hydroxyl group of serine or threonine residues, while OGA cleaves the GlcNAc from the modified protein^23^. Dysregulation of O-GlcNAcylation is associated with a variety of human diseases, including cancer, diabetes and neurodegenerative diseases. Therefore, emerging evidence suggests that protein O-GlcNAcylation would be a promising therapeutic target^23,24^.

Silva et al. investigated whether O-GlcNAc affects the inflammatory response to LPS induced systemic inflammation in mice, using glucosamine or thiamet-G to cause an acute increase in O-GlcNAcylation^17^. They demonstrated that increased O-GlcNAc attenuated the blood levels of proinflammatory cytokines and suppressed leukocyte infiltration in lungs and leukocyte migration to the peritoneal cavity. Glucosamine also decreased the production of proinflammatory cytokines in LPS induced in vitro cultures of mouse primary bone marrow macrophages and mouse macrophage cell line RAW264.7, probably by mechanisms that involved reduction of O-GlcNAc-modified NF-kB p65 subunit and hence, NF-kB activity. These findings were in accordance with our current and previously reported data^21^. Furthermore, several NAG derivatives with different 1-hydroxyl substitutions have been proven as OGA inhibitors which could lead to an accumulation of protein O-GlcNAc^16,25–27^. As such, we hypothesize that NAG, BNAG1 and BNAG2 might suppress immune response through the inhibition of OGA, and the reason why BNAG1 displays better activity than BNAG2 or NAG is due to its 1-deoxygenation. It is apparent that this hypothesis needs more detailed experimental evidence in the future research.

In addition to the mechanistic issue, further studies are mandatory to provide the necessary information on whether BNAG1 and BNAG2 are potential candidates of a viable therapeutic strategy to target inflammation in a variety of pathologies, including but not restricted to joint damages^6,7^, inflammatory bowel disease^8^, autoimmune diseases^9^, and viral respiratory infections^10,11^. While our experimental data indicated that BNAG1 was more effective than NAG or BNAG2, it would be interesting to investigate if it is also more powerful than glucosamine or other NAG derivatives mentioned above. On the other hand, NAG was reported to be a low toxic compound, and after 20 g intravenous injection its half-life in blood was 220 min^28^. In this regard, it is necessary to investigate the pharmacokinetics using radiolabeled methods^29^ and toxicity^30^ of BNAG1 and BNAG2 in the future.

In conclusion, this is the first report on the synthesis and possible biomedical application of two bi-oxygenated NAG derivatives. BNAG1 and BNAG2 were successfully prepared, and we demonstrated that BNAG1 had the highest activities than BNAG2 or NAG against LPS induced inflammatory response both in mice and in vitro macrophage cultures. However, further studies are needed to better clarify the exact mechanisms by which BNAG1 and BNAG2 mediate their effects and the potential application of these NAG derivatives, especially BNAG1 in the treatment of certain inflammation-related diseases.

## Methods

### Synthesis of BNAG1 and BNAG2

The synthetic routes of BNAG1 and BNAG2 were shown in Figs. 8 and 9, respectively. The procedures for their full synthesis were presented in Supplemental materials. The starting chemicals NAG for BNAG1 and N-((4aR,6S,7R,8R,8aS)-6-(Benzyloxy)-8-hydroxy-2-phenylhexahydropyrano[3,2-d][1,3]dioxin-7-yl)acetamide (CAS No. 13343–63-0) for BNAG2 were purchased from Thermo Fisher Scientific (Pittsburgh, PA, USA) and Ambeed (Arlington Hts, IL, USA), respectively.

### Statement

All experimental procedures were approved by the Ethics Committee on Animal Research of Medical School at UVa (Protocol Title: Fullerol based treatment for osteoarthritis repair; Protocol Number: 3933–06-21), and are in accordance with the Guidelines of the National Council for Animal Experimentation Control (CONCEA). All methods are reported in accordance with ARRIVE guidelines.

### Experimental animal model^17^

C57BL6/J mice (Male, 8 to 10-week-old, 20–25 g) purchased from Charles River were used in this study. Mice were kept in the animal facility of the School of Medicine, University of Virginia (UVa). Systemic inflammatory response to LPS (Escherichia coli 0111:B4, Sigma Chemical Co., St. Louis, MO, United States) challenge in mice was induced with a single dose (10 mg/Kg, intraperitoneal injection) of LPS, 30 min after intravenous injection of NAG or BNAG1 or BNAG2 or the vehicle (saline) with anesthesia induced and maintained by isoflurane. Two doses (200 mg/kg body weight and 300 mg/kg body weight) were tested for NAG, BNAG1 and BNAG2. Animals without any treatment were used to obtain the baseline data. Six hours after LPS treatment, all mice were euthanized by carbon dioxide and different inflammation assays were performed as described below. A total of 40 animals were randomly divided into 5 groups (8 mice/group): non-treatment (NT); 10 mg/Kg LPS plus saline (LPS); 10 mg/Kg LPS plus NAG (LPS + NAG); 10 mg/Kg LPS plus BNAG1 (LPS + BNAG1); and 10 mg/Kg LPS plus BNAG2 (LPS + BNAG2). The experiment was duplicated.

### Myeloperoxidase activity assay^17^

Leukocyte migration to the lungs was evaluated using a myeloperoxidase kinetic-colorimetric assay. Tissue samples from each mouse were collected in 50 mM K_2_HPO_4_ buffer (pH 6.0) containing 13.72 mM hexadecyltrimethylammonium bromide and stored at − 80 °C until assayed. The samples were homogenized using a Tissue-Tearor, and homogenates were centrifuged (13,000 rpm, 2 min, 4 °C). Supernatants were assayed spectrophotometrically for myeloperoxidase activity at 450 nm on a microplate reader.

### Leukocyte migration to the peritoneal cavity

Peritoneal cells were collected from each mouse by lavage with ice-cold Dulbecco's modified Eagle's medium (DMEM, Gibco BRL, Gaithersburg, MD)^17^. The peritoneal cells were fixed in 70% ethanol overnight at 4 °C. Fixed cells were then centrifuged onto a 96 well plate with coverglass bottom (Thermo Fisher Scientific) at 1,500 rpm at 4 °C for 10 min, double-stained with Alexa Fluo® 568 phalloidin (Thermo Fisher Scientific) and 4′,6-diamidino-2-phenylindole (DAPI, Thermo Fisher Scientific) which shows the actin cytoskeleton and cell nucleus, respectively. Cell fluorescence was analyzed with an Agilent BioTek Cytation 5 Cell Imaging Multi-Mode Reader and cell number was quantified by the Gen5 software attached to the reader.

### WST-1 assay

The cell proliferation reagent 4-[3-(4-Iodophenyl)-2-(4-nitro-phenyl)-2H-5-tetrazolio]-1,3-benzene sulfonate (WST-1) is widely used for the non-radioactive, spectrophotometric quantification of cell proliferation and viability, using the 96-well-plate format^31–33^. WST-1 was also proposed as an useful tool to determine the superoxide production by NADPH oxidase of phagocytic cells neutrophils and monocytes^34^. In the present study, WST-1 assay was performed to assess the in vivo and in vitro activity of BNAGs against peritoneal cells. For in vivo assay, peritoneal cells were harvested from mice treated by LPS alone, LPS + NAG, LPS + BANG1, or LPS + BANG2. Cells were cultured for 24 h on a 96 well plate in basal medium (DMEM supplemented with 10% FBS and antibiotics) and in a humidified atmosphere of 5% carbon dioxide at 37 °C. For in vitro assay, peritoneal cells were isolated from mice without any treatment, grew in basal medium on a 96 well plate, and treated with 100 ng/mL LPS for 24 h after addition of BNAGs and NAG at various doses for 0.5 h. After removal of the supernatant, 150 µL new medium was added into each cell. Then 15 µL WST-1 (Thermo Fisher Scientific) was added and incubated with cells for 3 h in dark with a humidified atmosphere of 5% carbon dioxide at 37 °C. The optical density (OD) value of each sample was determined at 450 nm on a microplate reader.

### Determination of cytokines Levels^33^

IL-6 and TNF α concentrations in the serum from mice or culture medium from primary peritoneal macrophages were determined using the enzyme-linked immunosorbent assay (ELISA) kits (Fisher Scientific Co.) according to the manufacturer’s instructions. Briefly, the serum from each animal or culture medium from each cell sample was collected and mixed with different assay reagents of each assay kit step by step. The resultant solutions were read at 450 nm on a microplate reader, and IL-6 and TNF α concentrations were calculated from the corresponding standard curves, respectively.

### Statistical analysis

All data was reported as mean ± SD, and statistical analysis was performed by the GraphPad Prism program. Statistical significance was determined by t tests (two-tailed) for two groups or ANOVA (with Dunnett’s multiple comparisons test) for three or more groups. In each figure, a selected control group was compared with the other samples individually. If the obtained *p* value < 0.05, the difference was considered as significant (*).

### Supplementary Information

Supplementary Information.

## Data Availability

The data presented in this study are available on request from the corresponding author.

## References

[1] Netea MG, Balkwill F, Chonchol M, Cominelli F, Donath MY, Giamarellos-Bourboulis EJ, Golenbock D, Gresnigt MS, Heneka MT, Hoffman HM (2017). A guiding map for inflammation. Nat Immunol.

[2] Furman D, Campisi J, Verdin E, Carrera-Bastos P, Targ S, Franceschi C, Ferrucci L, Gilroy DW, Fasano A, Miller GW (2019). Chronic inflammation in the etiology of disease across the life span. Nat Med.

[3] Wang RX, Zhou M, Ma HL, Qiao YB, Li QS (2021). The role of chronic inflammation in various diseases and anti-inflammatory therapies containing natural products. ChemMedChem.

[4] Ngum JA, Tatang FJ, Toumeni MH, Nguengo SN, Simo USF, Mezajou CF, Kameni C, Ngongang NN, Tchinda MF, Dongho Dongmo FF (2023). An overview of natural products that modulate the expression of non-coding RNAs involved in oxidative stress and inflammation-associated disorders. Front Pharmacol.

[5] Chen JK, Shen CR, Liu CL (2010). N-acetylglucosamine: production and applications. Mar Drugs.

[6] Azuma K, Osaki T, Wakuda T, Tsuka T, Imagawa T, Okamoto Y, Minami S (2012). Suppressive effects of N-acetyl-D-glucosamine on rheumatoid arthritis mouse models. Inflammation.

[7] Richter J, Capkova K, Hribalova V, Vannucci L, Danyi I, Maly M, Fiserova A (2014). Collagen-induced arthritis: severity and immune response attenuation using multivalent N-acetyl glucosamine. Clin Exp Immunol.

[8] Salvatore S, Heuschkel R, Tomlin S, Davies SE, Edwards S, Walker-Smith JA, French I, Murch SH (2000). A pilot study of N-acetyl glucosamine, a nutritional substrate for glycosaminoglycan synthesis, in paediatric chronic inflammatory bowel disease. Aliment Pharmacol Ther.

[9] Grigorian A, Araujo L, Naidu NN, Place DJ, Choudhury B, Demetriou M (2011). N-acetylglucosamine inhibits T-helper 1 (Th1)/T-helper 17 (Th17) cell responses and treats experimental autoimmune encephalomyelitis. J Biol Chem.

[10] Hassan AE (2021). An observational cohort study to assess N-acetylglucosamine for COVID-19 treatment in the inpatient setting. Ann Med Surg (Lond).

[11] Marchetti M, De Berardis B, Bigioni I, Mariano A, Superti F, Scotto d'Abusco A (2023). In vitro antiviral and anti-inflammatory activities of n-acetylglucosamine: development of an alternative and safe approach to fight viral respiratory infections. Int J Mol Sci.

[12] Lee SU, Li CF, Mortales CL, Pawling J, Dennis JW, Grigorian A, Demetriou M (2019). Increasing cell permeability of N-acetylglucosamine via 6-acetylation enhances capacity to suppress T-helper 1 (TH1)/TH17 responses and autoimmunity. PLoS ONE.

[13] Morrison ZA, Eddenden A, Subramanian AS, Howell PL, Nitz M (2022). Termination of Poly-N-acetylglucosamine (PNAG) polymerization with N-Acetylglucosamine analogues. ACS Chem Biol.

[14] Choi SY, Ahn YR, Lee EB, Yu MJ, Lee JR (2022). Expression of a RhoA-specific guanine nucleotide exchange factor, p190RhoGEF, in mouse macrophages negatively affects M1 polarization and inflammatory responses. Front Immunol.

[15] Blunden S, Wallace T (2003). Tin in canned food: a review and understanding of occurrence and effect. Food Chem Toxicol.

[16] Kiss M, Timari I, Barna T, Meszaros Z, Slamova K, Bojarova P, Kren V, Hayes JM, Somsak L (2022). 2-Acetamido-2-deoxy-d-glucono-1,5-lactone sulfonylhydrazones: synthesis and evaluation as inhibitors of human OGA and HexB enzymes. Int J Mol Sci.

[17] Silva JF, Olivon VC, Mestriner F, Zanotto CZ, Ferreira RG, Ferreira NS, Silva CAA, Luiz JPM, Alves JV, Fazan R (2019). Acute increase in O-GlcNAc improves survival in mice with LPS-induced systemic inflammatory response syndrome. Front Physiol.

[18] Hwang JS, Kim KH, Park J, Kim SM, Cho H, Lee Y, Han IO (2019). Glucosamine improves survival in a mouse model of sepsis and attenuates sepsis-induced lung injury and inflammation. J Biol Chem.

[19] D'Avino D, Cerqua I, Ullah H, Spinelli M, Di Matteo R, Granato E, Capasso R, Maruccio L, Ialenti A, Daglia M (2023). Beneficial effects of astragalus membranaceus (Fisch.) bunge extract in controlling inflammatory response and preventing asthma features. Int J Mol Sci.

[20] Li D, Yang L, Wang W, Song C, Xiong R, Pan S, Li N, Geng Q (2023). Eriocitrin attenuates sepsis-induced acute lung injury in mice by regulating MKP1/MAPK pathway mediated-glycolysis. Int Immunopharmacol.

[21] Le Q, Zhang Z, Sun D, Cui Q, Yang X, Hassan AE (2023). Anti-inflammatory activities of two new deoxygenated N-acetyl glucosamines in lipopolysaccharide-activated mouse macrophage RAW264.7 cells. Heliyon.

[22] Yang X, Qian K (2017). Protein O-GlcNAcylation: emerging mechanisms and functions. Nat Rev Mol Cell Biol.

[23] Paneque A, Fortus H, Zheng J, Werlen G, Jacinto E (2023). The hexosamine biosynthesis pathway: regulation and function. Genes (Basel).

[24] Zhu Y, Hart GW (2021). Targeting O-GlcNAcylation to develop novel therapeutics. Mol Aspects Med.

[25] Hattie M, Cekic N, Debowski AW, Vocadlo DJ, Stubbs KA (2016). Modifying the phenyl group of PUGNAc: reactivity tuning to deliver selective inhibitors for N-acetyl-D-glucosaminidases. Org Biomol Chem.

[26] Macauley MS, Whitworth GE, Debowski AW, Chin D, Vocadlo DJ (2005). O-GlcNAcase uses substrate-assisted catalysis: kinetic analysis and development of highly selective mechanism-inspired inhibitors. J Biol Chem.

[27] Kiss M, Szabo E, Bocska B, Sinh LT, Fernandes CP, Timari I, Hayes JM, Somsak L, Barna T (2021). Nanomolar inhibition of human OGA by 2-acetamido-2-deoxy-d-glucono-1,5-lactone semicarbazone derivatives. Eur J Med Chem.

[28] Levin RM, Krieger NN, Winzler RJ (1961). Glucmsumine and acetylglucosamine tolerance in man. J Lab Clin Med.

[29] Fujiwara T, Kubota K, Tada M, Itoh M, Sato T, Iwata R, Sato K, Takahashi J, Abe Y, Fukuda H (1992). N-(F-18)-fluoroacetyl-D-glucosamine: a new positron labeled pharmaceutical for cancer study. Tohoku J Exp Med.

[30] Lee KY, Shibutani M, Takagi H, Arimura T, Takigami S, Uneyama C, Kato N, Hirose M (2004). Subchronic toxicity study of dietary N-acetylglucosamine in F344 rats. Food Chem Toxicol.

[31] Berridge MV, Herst PM, Tan AS (2005). Tetrazolium dyes as tools in cell biology: new insights into their cellular reduction. Biotechnol Annu Rev.

[32] Jurado S, Pares A, Peris P, Combalia A, Monegal A, Guanabens N (2022). Bilirubin increases viability and decreases osteoclast apoptosis contributing to osteoporosis in advanced liver diseases. Bone.

[33] Pei Y, Cui F, Du X, Shang G, Xiao W, Yang X, Cui Q (2019). Antioxidative nanofullerol inhibits macrophage activation and development of osteoarthritis in rats. Int J Nanomed.

[34] Tan AS, Berridge MV (2000). Superoxide produced by activated neutrophils efficiently reduces the tetrazolium salt, WST-1 to produce a soluble formazan: a simple colorimetric assay for measuring respiratory burst activation and for screening anti-inflammatory agents. J Immunol Methods.

