# Supplementary material for: Synthesis and anti-inflammatory activities of two new N-acetyl glucosamine derivatives
Source: Sci Rep. 2024 May 14;14:11079. doi: 10.1038/s41598-024-61780-2 (PMC11094000; doi:10.1038/s41598-024-61780-2)
Supplement: Supplementary file 1 — Supplementary Information. [file 41598_2024_61780_MOESM1_ESM.pdf]

Synthesis and anti-inflammatory activities of two new N-acetyl glucosamine derivatives

Zhichang Zhang[1,3], Weicheng Wang[1], Peng Xu[2], Quanjun Cui[1], Xinlin Yang*[,1], Ameer E. Hassan*[,4]

[1] Dept of Orthopaedic Surgery and [2] Dept of Pathology, University of Virginia, 22903, USA
[3] Dept of Orthopaedic Surgery, The First Affiliated Hospital of Xinxiang Medical University, Wehui, 453100, Henan, China
[4] Department of Neuroscience, Valley Baptist Medical Center, 78550, USA

Correspondence should be addressed to both

Xinlin Yang, xy3c@virginia.edu
Department of Orthopaedic Surgery
University of Virginia School of Medicine
450 Ray C. Hunt Drive, Charlottesville, Virginia 22908

Ameer E. Hassan, ameerehassan@gmail.com,
Department of Neuroscience,
Valley Baptist Medical Center,
2101 Pease St, Harlingen, TX, 78550, USA

1. Procedure for BNAG1 Synthesis (See Figure 1 in the main text)

1.1. General Procedure for the Preparation of Compound 2

To a solution of compound **1** (200 g) in AcCl (600 mL) was stirred at 30°C for 48 hrs. The reaction mixture was diluted with DCM (2 L) and poured into ice water (2 L). The mixture was extracted with DCM (1 L x 3). The combined organic layers were washed with saturated aqueous $NaHCO_3$ (1 L x 2) and brine (800 mL x 3). The combined organic layer was dried over anhydrous Na_2SO_4, then filtered and concentrated *in vacuo*. The residue was purified by column chromatography on silica gel (PE: EA, 1:0 to 1:1) to afford compound **2** (162.6 g, 49 %) as light yellow solid. **TLC**: PE: EA =1:2, I_2; Rf (compound 2) = 0.5.

1.2. General Procedure for the Preparation of Compound 3

To a suspension of compound **2** (162.5 g, 442.3 mmol, 1.0 eq) in toluene (1.6 L) was added AIBN (7.3 g, 44.2 mmol, 0.1 eq) and tri-*n*-butyltin hydride (193.2 g, 663.5 mmol, 1.5 eq). The reaction mixture was stirred at 118 ^{o}C under N_2 for 16 hrs. The reaction mixture was poured into KF aqueous solution (2N, 1.6 L) and stirred for 1 hr. The mixture was filtered, and the filtrate was extracted with DCM (1 L x 3). The combined organic layers were washed with brine (900 mL x 3). The organic layer was dried with anhydrous Na_2SO_4, filtered and concentrated *in vacuo*. The residue was purified by column chromatography on silica gel (PE: EA, 3:1 to 0:1) to afford compound **3** (134.0 g, 91%) as white solid. **TLC**: PE: EA =0:1, UV; Rf (compound 2) = 0.7, Rf (compound 3) = 0.4.

1.3. General Procedure for the Preparation of Compound 4

To a solution of compound **3** (134.0 g, 404.4 mmol, 1.0 eq) in methanol (1.4 L) was added $NaOCH_3$ (132 g, 2.42 mol, 6.0 eq). The mixture was stirred at rt for 16 hrs under N_2. The reaction mixture was neutralized. The mixture was filtered and the filtrate was concentrated *in vacuo* to afford crude compound **4** (154 g) as light yellow gum, which was used in next step without further purification.

1.4. General Procedure for the Preparation of Compound 6

To a suspension of compound **4** (98.0 g, 407.6 mmol, 1.0 eq) in benzaldehyde (**5**, 950 mL) was added $ZnCl_2$ (130.0 g, 955.1 mmol, 2.3 eq). The reaction mixture was stirred at rt under N_2 for 16 hrs. Water (800 mL) and petroleum ether (PE, 500 mL) was

added. The mixture was filtered and the filter cake was washed with PE, and dried to afford compound **6** (85.0 g, 88% for 2 steps) as white solid.

1.5. General Procedure for the Preparation of Compound 8

To a suspension of compound **6** (102.0 g, 347.7 mmol, 1.0 eq) in acetonitrile (1.8 L) were added DMAP (127.4 g, 1043.1 mmol, 5.0 eq) and compound **7** (90.0 g, 521.6 mmol, 1.5 eq). The reaction mixture was stirred at rt under N_2 for 3 hrs. The mixture was filtered. The filtrate was washed with aq HCl (1M, 600 mL x 3) and the organic phase was concentrated. The residue was triturated with PE: EA (5:1) to afford compound **8** (98.0 g, 68%) as off-white solid. **TLC**:PE: EA =1:2, UV; Rf (compound **6**) = 0.15, Rf (compound **8**) =0.6.

1.6. General Procedure for the Preparation of Compound 9

To a suspension of compound **8** (78.0 g, 181.7 mmol, 1.0 eq) in toluene (780 mL) was added AIBN (9.0 g, 54.5 mmol, 0.3 eq) and tri-*n*-butyltin hydride (264.6 g, 908.5 mmol, 5.0 eq). The reaction mixture was stirred at 118 ^{o}C under N_2 for 16 hrs. The reaction mixture was poured into aqueous KF solution (2N, 1.6 L) and the mixture was stirred for 1 hr. Then the mixture was filtered and the filtrate was extracted with DCM (800 mL x 3). The combined organic layers were washed with brine (900 mL x 3), and concentrated *in vacuo*. The residue was purified by column chromatography on silica gel (PE: EA, 3:1 to 0:1) to afford compound **9** (28.8 g, 57 %) as white solid. **TLC**: PE: EA =1:2, $I_{2;}$ Rf (compound **8**) = 0.5, Rf (compound **9**) = 0.3.

1.7. General Procedure for the Preparation of Compound BNAG1

To a suspension of compound **9** (12.0 g, 42.3 mmol, 1.0 eq) in acetic acid (150 mL) was added water (300 mL). The reaction mixture was stirred at 95 ^{o}C under N_2 for 1 hr. The reaction mixture was concentrated under reduced pressure. The residue was dissolved in toluene (100 mL) and the mixture was washed with water (30 mL). The organic phase was concentrated and the residue was purified by column chromatography on silica gel (DCM: MeOH, 50:1 to 15:1) to afford compound **BNAG1** (7.0 g, 85 %) as white solid. LC-MS: 190.20 $[M+1]^{+}$. ^{1}H NMR (400 MHz, CD_3OD) δ 3.94 – 3.79 (m, 3H), 3.61 – 3.52 (m, 1H), 3.48 – 3.41 (m, 1H), 3.05 – 2.95 (m, 2H), 2.25 – 2.18 (m, 1H), 1.90 (s, 3H), 1.37 (q, *J* = 11.7 Hz, 1H). ^{13}C NMR (100 MHz, DMSO-d6): δ 169.3, 83.4,

69.64, 65.0, 61.9, 45.2, 22.8. Anal. Calcd for $C_8H_{15}NO_4$: C, 50.78; H, 7.99; N, 7.40. Found: C, 50.58; H, 7.88; N, 7.21. $[\alpha]^{25}_D$ 4.88 ° (c = 1.0, methanol).

2. Procedure for BNAG2 Synthesis (See Figure 2 in main text)

2.1. General Procedure for the Preparation of Compound 11

To a solution of compound **10** (59 g, 147.7 mmol) in pyridine (413 mL) was added acetic anhydride (60 mL) at room temperature (25~30 °C). After addition, the resulting mixture was stirred at room temperature (25~30 °C) overnight. The reaction was monitored by TLC. After compound **10** consumed completely, the reaction mixture was concentrated under reduced pressure at 50 °C. The residue was co-concentrated under reduced pressure with toluene (300 mL x 4) to afford compound **11** (64 g, 98%) as a white solid.

11: LC-MS: 442.3 $[M+1]^+$. ^1H NMR (400 MHz, DMSO-d_6) δ 8.02 (d, *J* = 9.2 Hz, 1H), 7.44 – 7.25 (m, 10H), 5.60 (s, 1H), 5.17 (dd, *J* = 10.7, 8.8 Hz, 1H), 4.80 (d, *J* = 3.6 Hz, 1H), 4.71 (d, *J* = 12.3 Hz, 1H), 4.51 (d, *J* = 12.3 Hz, 1H), 4.21 – 4.10 (m, 2H), 3.83 – 3.71 (m, 3H), 1.94 (s, 3H), 1.79 (s, 3H).

2.2. General Procedure for the Preparation of compound 12

A solution of compound **11** (64 g) in 80% HOAc (1.9 L HOAc in 500 mL water) was heated and stirred at 65~70 °C for 3 hours. The reaction was monitored by LC-MS. After compound **11** consumed completely, the reaction mixture was concentrated under reduced pressure at 50 °C. The residue was co-concentrated under reduced pressure with toluene (300 mL x 3) to afford compound **12** (58.8 g) as a solid.

12: LC-MS: 354.3 $[M+1]^+$. ^1H NMR (400 MHz, DMSO-d_6) δ 7.76 (d, *J* = 9.3 Hz, 1H), 7.39 (d, *J* = 6.9 Hz, 2H), 7.36 – 7.30 (m, 2H), 7.30 – 7.25 (m, 1H), 5.34 (s, 1H), 4.99 (dd, *J* = 11.1, 8.9 Hz, 1H), 4.72 – 4.64 (m, 2H), 4.43 (d, *J* = 12.2 Hz, 1H), 3.94 (ddd, *J* = 11.1, 9.4, 3.5 Hz, 1H), 3.60 (d, *J* = 9.8 Hz, 2H), 3.51 (d, *J* = 8.5 Hz, 2H), 1.92 (d, *J* = 2.1 Hz, 3H), 1.77 (s, 3H).

2.3. General Procedure for the Preparation of compound 13

To a solution of compound **12** (58.8 g, 166.4 mmol) in pyridine (420 mL) under N_2 atmosphere at -10°C, was added sulfuryl chloride (56.2 g, 416 mmol) dropwise below 0°C. The reaction mixture was stirred at 0 °C for 30 minutes, then warmed up to room temperature (25~30 °C) and stirred for another 3 hours. The reaction was monitored by LC-MS. After compound **12** consumed completely, the reaction mixture was

concentrated under reduced pressure at 50 ^{o}C, then toluene (300 mL) was added to the residue and co-concentrated to remove pyridine. To the residue was added chloroform (500 mL), water (350 mL) and chloroform (300 mL). After separation, the organic layer was washed with water (300 mL x 3), dried over anhydrous Na_2SO_4 and concentrated. Toluene (200 mL) was added and co-concentrated. The residue was triturated with PE/EA (10/1, 420 mL) at room temperature for 4 hours to afford compound **13** (39.8 g) as a yellow solid.

13: LC-MS: 390.2 $[M+1]^+$. ^1H NMR (400 MHz, DMSO-d_6) δ 8.05 (d, J = 8.5 Hz, 1H), 7.40 – 7.32 (m, 4H), 7.30 (dd, J = 5.7, 2.8 Hz, 1H), 5.12 (dd, J = 11.6, 3.4 Hz, 1H), 4.89 (d, J = 3.6 Hz, 1H), 4.78 – 4.70 (m, 2H), 4.49 (d, J = 12.0 Hz, 1H), 4.34 (td, J = 8.3, 4.3 Hz, 1H), 4.30 – 4.24 (m, 1H), 3.78 (dd, J = 11.2, 4.6 Hz, 1H), 3.64 (dd, J = 11.2, 7.9 Hz, 1H), 2.00 (s, 3H), 1.80 (s, 3H).

2.4. General Procedure for the Preparation of compound 14

To a solution of compound **13** (39.8 g, 101.98 mmol) in toluene (800 mL) under N_2 atmosphere was added *n*-Bu_3SnH (59.2 g, 204.1 mmol) and AIBN (8.4 g, 51.2 mmol). The mixture was heated to 110 ^{o}C (inner temperature) and stirred for 8 hours. The reaction was monitored by LCMS. Mono-Cl intermediate remained by LCMS (*m/z*: 356). Then *n*-Bu_3SnH (0.5 eq) and AIBN (0.1 eq) were added every 5 hours until no mono-Cl intermediate remained by LCMS. LCMS also showed small amount of de-acetyl product (compound **15**).

The reaction mixture was concentrated at 50 ^{o}C, then co-concentrated with petroleum ether (100 mL x 3). The residue was triturated with petroleum ether (200 mL) at -30°C for 1 hour and viscous solid formed. The supernatant was collected and the viscous solid re-triturated with petroleum ether (200 mL) at -30 ^{o}C for 1 hour. The trituration process repeated 3~4 times until sandy solid obtained. The solid was filtered to afford compound **14** (40 g), meanwhile, the combined supernatant was purified by flash column (PE/EA= 5/1~1/1) to afford another batch of compound **14** (2 g). Over all, compound **14** (42 g) obtained. Theoretical quantity: 32.8 g, thus the yield exceeded 100%. Theoretical quantity used for next step calculation.

14: is a crude product without characterization.

2.4. General Procedure for the Preparation of compound 15

To a 2 L three-neck flask was charged dry methanol (820 mL) and cooled to 0°C under N_2 atmosphere, then sodium methanolate (11.1 g, 205.6 mmol) was added and stirred until clear solution formed. Compound **14** (32.8 g, 102.1 mmol, theoretical quantity) dissolved in dry methanol (100 mL) was added into above solution dropwise at 0 °C. After addition, the mixture was warmed up to room temperature (25~30 °C) and stirred for 2 hours. The reaction was monitored by LC-MS. After compound **14** consumed completely, the mixture was concentrated under reduced pressure at 50 °C. Water (100 mL) was added, extracted with ethyl acetate (100 mL x 3). The organic layers were dried over anhydrous Na_2SO_4 and concentrated. The crude was purified by flash column (PE/EA, 5/1~2/1~1/3) to afford compound **15** (19 g, 67%).

15: LC-MS: 280.25 $[M+1]^+$. ^1H NMR (400 MHz, DMSO-*d*$_6$) *δ* 7.74 (d, *J* = 8.1 Hz, 1H), 7.36 – 7.29 (m, 4H), 7.28 – 7.23 (m, 1H), 4.69 (d, *J* = 3.5 Hz, 1H), 4.64 (d, *J* = 6.0 Hz, 1H), 4.59 (d, *J* = 12.6 Hz, 1H), 4.39 (d, *J* = 12.7 Hz, 1H), 3.84 (t, *J* = 9.2 Hz, 1H), 3.71 (dt, *J* = 10.7, 5.4 Hz, 1H), 3.54 (ddd, *J* = 10.4, 8.1, 3.5 Hz, 1H), 1.92 (ddd, *J* = 12.8, 4.8, 2.2 Hz, 1H), 1.81 (s, 3H), 1.19 (q, *J* = 11.7 Hz, 1H), 1.08 (d, J = 6.2 Hz, 3H).

2.5. General Procedure for the Preparation of compound BNAG2

To a solution of compound **15** (8 g, 28.64 mmol) in methanol (800 mL) was added 10% Pd/C (40 g, 50% water content). The reaction was stirred at room temperature (25~30°C) overnight. The reaction mixture was monitored by LC-MS. After compound **15** consumed completely, the mixture was filtered through a pad of Celite, and washed with MeOH/H_2O (1/1, 400 mL x 3). The filtrate was concentrated and re-dissolved in methanol (100 mL), then filtrated through Celite. The filtrate was concentrated and co-concentrated with methanol (50 mL x 3) to afford 5 g white solid. The solid was triturated with ethyl acetate (50 mL) to afford compound **BNAG2** (4.0 g, 74%). LC-MS: 190.20 $[M+H]^+$. ^1H NMR (400 MHz, DMSO-*d*$_6$): *δ* 7.55 (s, 1 H), 6.24 (s, 1 H), 4.89 (s, 1 H), 4.54 (s, 1 H), 3.98–3.95 (m, 1 H), 3.66–3.62 (m, 1 H), 3.45–3.40 (m, 1 H), 1.87–1.83 (m, 1 H), 1.79 (s, 3 H), 1.13–1.07 (m, 1 H) 1.03 (s, 3 H). ^{13}C NMR (100 MHz, DMSO-d6): δ 170.0, 91.7, 64.4, 62.8, 56.3, 42.9, 23.2, 21.5. Anal. Calcd for $C_8H_{15}NO_4$: C, 50.78; H, 7.99; N, 7.40. Found: C, 50.60; H, 8.24; N, 7.32. $[\alpha]^{25}_D$ -23.8 ° (c = 1.0, methanol).
